# Impact of integrating paper-based pulmonary function test case group discussions into flipped classroom on residents’ COPD grading assessment competency

**DOI:** 10.3389/fmed.2025.1671616

**Published:** 2026-01-07

**Authors:** Xingren Liu, Hang Long

**Affiliations:** Department of Pulmonary and Critical Care Medicine, Sichuan Academy of Medical Sciences and Sichuan Provincial People’s Hospital, School of Medicine, University of Electronic Science and Technology of China, Chengdu, Sichuan, China

**Keywords:** flipped classroom, case-based discussion, pulmonary function test, chronic obstructive pulmonary disease, medical residents

## Abstract

**Background:**

The flipped classroom model combined with case-based learning has emerged as a promising educational approach in medical residency training. However, the specific impact of integrating paper-based pulmonary function test case discussions on residents’ ability to accurately assess COPD severity remains underexplored.

**Objective:**

To investigate the effect of incorporating paper-based pulmonary function test case group discussions within a flipped classroom framework on residents’ competency in COPD grading assessment, comprehensive examination performance, and self-efficacy.

**Methods:**

This single-center, retrospective cohort study analyzed 100 residents who completed respiratory medicine rotations between 2022 and 2024. Participants were divided into an intervention group (*n* = 50) receiving flipped classroom instruction with paper-based case discussions and a control group (*n* = 50) receiving traditional didactic teaching. Outcomes included COPD grading accuracy rates, comprehensive examination scores, and self-efficacy levels measured by the Self-Efficacy for Professional Tasks (SEPT) scale.

**Results:**

The intervention group demonstrated significantly higher COPD grading accuracy rates both immediately post-instruction (86.4% vs. 68.2%, *p* < 0.001) and at one-month follow-up (82.6% vs. 64.8%, *p* < 0.001). Comprehensive examination scores were superior in the intervention group (88.2 ± 6.4 vs. 78.6 ± 8.2, *p* < 0.001), as were self-efficacy scores (4.2 ± 0.5 vs. 3.4 ± 0.6, *p* < 0.001). Teaching satisfaction ratings were also higher in the intervention group (4.6 ± 0.4 vs. 3.8 ± 0.7, *p* < 0.001).

**Conclusion:**

Integrating paper-based pulmonary function test case discussions into flipped classroom teaching significantly enhances residents’ COPD grading assessment competency, examination performance, and professional self-efficacy. This innovative pedagogical approach merits wider implementation in respiratory medicine residency training programs.

## Introduction

Chronic obstructive pulmonary disease (COPD) represents a major global health challenge, characterized by persistent respiratory symptoms and airflow limitation due to airway and alveolar abnormalities (1). Accurate assessment and grading of COPD severity using spirometric data remains fundamental to clinical decision-making, yet studies indicate that many residents struggle with interpreting pulmonary function tests and applying classification criteria correctly (2). The Global Initiative for Chronic Obstructive Lung Disease (GOLD) criteria, which utilize post-bronchodilator forced expiratory volume in one second (FEV1) to forced vital capacity (FVC) ratios alongside symptomatic assessment, require sophisticated clinical reasoning skills that traditional didactic teaching methods may inadequately develop (3).

The flipped classroom model has gained considerable traction in medical education as an innovative pedagogical approach that inverts traditional teaching structures (4). In this model, learners engage with foundational content independently before class, allowing face-to-face sessions to focus on active learning, problem-solving, and knowledge application (5). Recent meta-analyses have demonstrated that flipped classroom approaches in health professions education lead to improved learning outcomes, enhanced critical thinking skills, and greater learner satisfaction compared to traditional lecture-based instruction (6, 7). The model appears particularly effective for developing higher-order cognitive skills essential for clinical practice, including data interpretation, clinical reasoning, and decision-making (8).

Small group case-based discussions represent a powerful complement to flipped classroom methodology, particularly in clinical education contexts (9). By presenting authentic clinical scenarios, case-based learning facilitates the integration of theoretical knowledge with practical application, promotes collaborative learning, and develops clinical reasoning skills (10). When applied to respiratory medicine education, case-based approaches using real patient data, including pulmonary function test results, have shown promise in improving diagnostic accuracy and clinical confidence (11). However, the specific use of paper-based materials, which allow tactile manipulation and annotation, may offer unique advantages over digital formats in promoting active engagement and retention (12).

Despite the growing adoption of flipped classroom and case-based learning approaches in medical education, significant gaps remain in our understanding of their effectiveness for specific clinical competencies. Most existing research has focused on undergraduate medical education or broad clinical skills, with limited investigation of specialized competencies in residency training (13). Furthermore, while digital learning materials predominate in contemporary medical education, the potential benefits of paper-based resources for complex data interpretation tasks remain underexplored. The integration of paper-based pulmonary function test reports in group discussions may facilitate deeper engagement with the material, promote collaborative analysis, and better simulate real-world clinical scenarios where physical documents remain common (14).

This study addresses these knowledge gaps by examining the impact of a novel educational intervention combining flipped classroom methodology with paper-based pulmonary function test case discussions on residents’ COPD grading competency. We hypothesized that residents exposed to this integrated approach would demonstrate superior accuracy in COPD severity assessment, improved overall clinical knowledge, enhanced self-efficacy, and greater satisfaction with their learning experience compared to those receiving traditional didactic instruction.

## Materials and methods

### Study design

This investigation employed a retrospective cohort design to evaluate the effectiveness of an innovative educational intervention in respiratory medicine residency training. The study was conducted at a tertiary care academic medical center with an established three-year internal medicine residency program enrolling approximately 40 residents per year. Data were collected from electronic educational records and assessment databases covering the period from January 1, 2022, to December 31, 2024. The study protocol received approval from the institutional review board, with a waiver of informed consent granted due to the retrospective nature and minimal risk design. All procedures adhered to ethical guidelines for educational research and followed the Strengthening the Reporting of Observational Studies in Epidemiology (STROBE) reporting standards.

### Study participants

The study population comprised internal medicine residents who completed their mandatory four-week respiratory medicine rotation during the specified timeframe. Inclusion criteria encompassed: (1) successful completion of the entire respiratory medicine rotation without interruption, (2) attendance at all scheduled educational sessions without documented absences exceeding 10% of total contact hours, (3) completion of all required assessments including pre-rotation baseline evaluation, immediate post-rotation assessment, and one-month follow-up evaluation, and (4) documented consent for retrospective analysis of educational data as part of standard residency program agreements. Exclusion criteria included: (1) incomplete assessment records with missing data exceeding 10% of required elements, (2) previous participation in similar educational interventions during earlier rotations or at other institutions, (3) concurrent enrollment in pulmonary medicine fellowship programs or possession of prior specialized respiratory medicine training, and (4) documented learning disabilities requiring specialized educational accommodations that might confound outcome assessment. From an initial pool of 168 eligible residents, 120 met all inclusion criteria. Following data quality review and verification of group assignment documentation, 100 residents with complete datasets were included in the final analysis. Participants were categorized into two groups based on the educational approach employed during their rotation period: the intervention group (*n* = 50) received instruction through the flipped classroom model enhanced with paper-based pulmonary function test case discussions, while the control group (*n* = 50) underwent traditional didactic teaching following the standard curriculum. Group assignment reflected natural variation in educational delivery methods as the program gradually transitioned from traditional to innovative teaching approaches during the study period. Specifically, residents who rotated earlier in the study period (e.g., 2022) received the traditional curriculum, while those who rotated later (e.g., 2023–2024) received the flipped classroom intervention. This retrospective cohort design was employed to evaluate the real-world implementation of the new educational strategy.

### Educational interventions

The intervention group participated in a carefully structured flipped classroom program designed to maximize active learning and clinical reasoning skill development. The pre-class preparation phase commenced 1 week before the rotation, with residents receiving access to a comprehensive online learning management system (Moodle platform). Required preparatory materials included: (1) video lectures totaling 180 min covering COPD pathophysiology, spirometry principles, and GOLD classification criteria, produced by respiratory medicine faculty and featuring interactive elements and self-assessment questions (Appendix A); (2) curated reading assignments from current GOLD reports and peer-reviewed literature, with emphasis on practical application of diagnostic criteria; (3) interactive online modules demonstrating spirometry interpretation techniques with immediate feedback mechanisms; and (4) formative assessments comprising (20 multiple-choice questions, Appendix B) designed to ensure foundational knowledge acquisition before face-to-face sessions. During in-class sessions, residents engaged in structured small group activities designed to promote collaborative learning and clinical reasoning. Groups of 5–6 residents were formed based on diverse backgrounds and experience levels to optimize peer learning. Each group received a portfolio of 8–10 authentic paper-based pulmonary function test reports representing various COPD phenotypes and severity levels. These materials included complete spirometry data with flow-volume loops, bronchodilator response assessments, and relevant clinical information. The paper format was deliberately chosen to facilitate annotation, comparison between cases, and collaborative analysis around a shared workspace. Group activities followed a structured protocol: (1) individual review and initial interpretation (15 min), (2) collaborative discussion and consensus building regarding COPD presence and severity grading (30 min), (3) completion of standardized assessment forms documenting FEV1/FVC ratios, percentage predicted values, and GOLD grade assignments with supporting rationale (20 min), and (4) facilitated discussion with expert faculty providing targeted feedback and clarification (25 min).

The control group received traditional didactic instruction following the established residency curriculum. This consisted of four 90-min lectures delivered by respiratory medicine faculty covering COPD epidemiology, pathophysiology, diagnostic approaches, and management principles. Lectures employed standard slide presentations with limited interactive elements. Pulmonary function test interpretation was taught through projected examples with instructor-led analysis. Large group discussions following lectures allowed for questions but provided limited opportunity for hands-on practice or peer collaboration. No pre-class preparation was required beyond general reading recommendations, and assessment occurred primarily through end-of-rotation examinations.

The total in-class session time for the intervention group was 90 min. The control group received 6 h of in-class lecture time. Although the intervention group’s formal in-class time was shorter, it was supplemented by 3 h of structured pre-class learning. The allocation and nature of the instructional time are compared in Table 1.

**Table 1:** Comparison of instructional time allocation between study groups.

Instructional component	Intervention group (*n* = 50)	Control group (*n* = 50)
Structured pre-class study	3 h (video lectures, readings, online modules)	Not formally assigned
Formal in-class time	90 min	6 h
Lecture/Content delivery	0 h	6 h
Small group case discussion	90 min	0 h
Faculty facilitation & feedback	(Integrated within case discussion)	(Limited Q&A within lectures)
Total structured learning time	4.5 h	6 h
Primary in-class focus	Active application, collaboration, and feedback	Passive content delivery

### Outcome measures and data collection

The primary outcome measure was COPD grading accuracy, assessed through standardized case-based evaluations administered at two time points: immediately following the educational intervention and at one-month follow-up. Each assessment comprised 10 unique pulmonary function test cases requiring interpretation and GOLD grade assignment. Cases were carefully selected to represent the full spectrum of COPD severity and included borderline scenarios requiring nuanced interpretation. Scoring criteria awarded full credit for correct GOLD grade assignment (GOLD 1–4) with appropriate identification of airflow limitation (FEV1/FVC < 0.70 post-bronchodilator) and partial credit for correct identification of airflow limitation with minor grading errors. Inter-rater reliability for scoring was established through duplicate grading of 20% of assessments, achieving a kappa coefficient of 0.920.

Secondary outcomes encompassed multiple dimensions of educational effectiveness. Comprehensive clinical knowledge was evaluated through a 100-item multiple-choice examination covering respiratory medicine topics, with 30 items specifically addressing COPD diagnosis, classification, and management. The examination underwent psychometric validation demonstrating appropriate difficulty indices and discrimination values. Self-efficacy for clinical tasks was measured using the validated Self-Efficacy for Professional Tasks (SEPT) instrument, comprising 23 items rated on a 5-point Likert scale addressing confidence in performing respiratory medicine-related clinical activities. The internal consistency of the SEPT scale in our study sample was excellent, with a Cronbach’s alpha coefficient of 0.913. Teaching satisfaction was assessed through a locally developed 15-item questionnaire evaluating various aspects of the educational experience including content relevance, teaching methods, faculty effectiveness, and perceived learning outcomes.

Baseline demographic and educational variables were extracted from residency program records, including age, gender, medical school background (domestic versus international), prior clinical experience, and academic performance metrics. These variables enabled assessment of group comparability and adjustment for potential confounders in multivariable analyses. Data collection followed standardized protocols with double data entry for critical variables and systematic verification procedures. Missing data, which affected less than 3% of outcome variables, were addressed through multiple imputation procedures to maintain statistical power and minimize bias.

### Statistical analysis

A post-hoc power analysis was conducted using G*Power 3.1 software. With a significance level (*α*) of 0.05 and an observed effect size (Cohen’s *d*) of 0.6 for the primary outcome, the achieved statistical power (1 − *β*) for the total sample size of 100 participants (50 cases in the intervention group and 50 cases in the control group) was 0.84, indicating adequate power to detect a clinically meaningful difference. Statistical analyses were performed using R software version 4.3.2 with appropriate packages for educational research applications. Descriptive statistics characterized participant demographics and baseline characteristics, with continuous variables presented as means with standard deviations or medians with interquartile ranges based on distribution assessment, and categorical variables expressed as frequencies with percentages. Between-group comparability was evaluated using independent samples *t*-tests for normally distributed continuous variables, Mann–Whitney *U* tests for non-parametric data, and chi-square tests for categorical variables. Primary outcome analysis compared COPD grading accuracy rates between intervention and control groups using independent samples *t*-tests, with effect sizes calculated using Cohen’s *d* to quantify the magnitude of differences. Secondary outcomes were analyzed using similar approaches, with Bonferroni correction applied to adjust for multiple comparisons. Repeated measures analysis of variance examined changes in accuracy over time and potential group-by-time interactions. Multivariable linear regression models were constructed to examine the independent association between educational intervention and outcomes while adjusting for potential confounders. Model building followed theoretical considerations, incorporating variables identified *a priori* as potentially influential: age, gender, medical school background, baseline academic performance, and prior respiratory medicine exposure. Model assumptions were verified through residual analysis, with appropriate transformations applied as necessary. Sensitivity analyses included propensity score matching to address potential selection bias, with 1:1 nearest neighbor matching achieving excellent balance across all covariates (standardized mean differences <0.1). All analyses employed two-tailed tests with statistical significance set at *p* < 0.05.

## Results

### Study flow and participant characteristics

**Figure 1:**
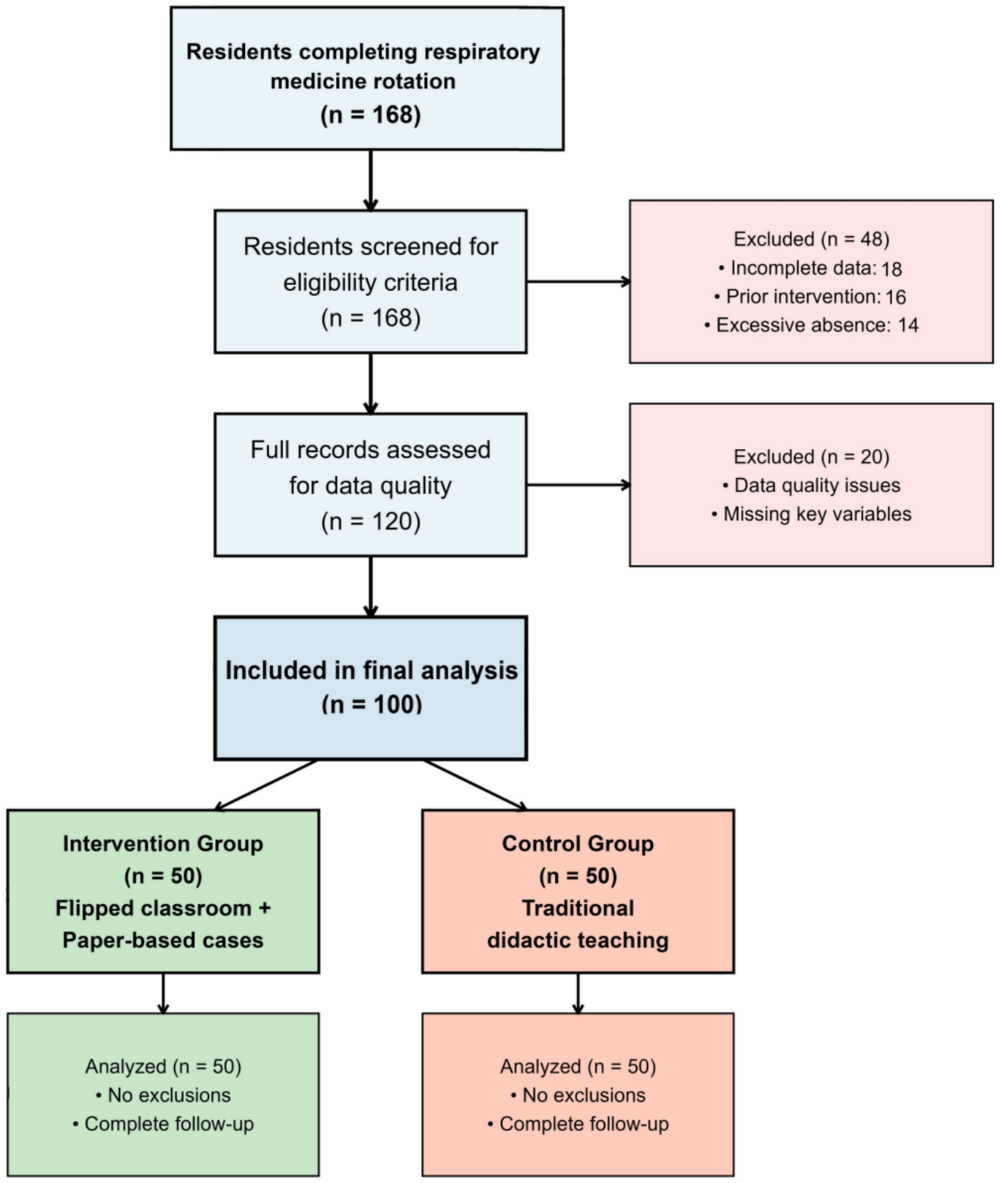
PRISMA-style flowchart depicting participant screening, eligibility assessment, and final group allocation.

The study screening process identified 168 potentially eligible residents who completed respiratory medicine rotations during the study period. Following application of inclusion and exclusion criteria, 100 residents were included in the final analysis, equally distributed between intervention (*n* = 50) and control (*n* = 50) groups (Figure 1). The most common reasons for exclusion included incomplete assessment data (*n* = 18), previous exposure to similar educational interventions (*n* = 16), and documented absences exceeding permitted thresholds (*n* = 14).

Baseline characteristics demonstrated excellent balance between study groups (Table 2). The mean age was 28.6 ± 2.3 years in the intervention group and 28.9 ± 2.5 years in the control group (*p* = 0.524). Both groups showed similar gender distributions (intervention: 54% female; control: 52% female, *p* = 0.841) and comparable proportions of international medical graduates (intervention: 32%; control: 36%, *p* = 0.673). Academic performance metrics, including United States Medical Licensing Examination Step 2 Clinical Knowledge scores and in-training examination percentiles, showed no significant differences. Prior exposure to respiratory medicine through elective rotations or research experiences was minimal and equally distributed between groups.

**Table 2:** Baseline demographic and educational characteristics of study participants.

Characteristic	Intervention group (*n* = 50)	Control group (*n* = 50)	*p*-value
Age, years (mean ± SD)	28.6 ± 2.3	28.9 ± 2.5	0.524
Female gender, *n* (%)	27 (54.0)	26 (52.0)	0.841
International medical graduate, *n* (%)	16 (32.0)	18 (36.0)	0.673
Education level, *n* (%)			0.812
MD only	38 (76.0)	37 (74.0)	
MD/PhD	7 (14.0)	8 (16.0)	
MD/MPH or other	5 (10.0)	5 (10.0)	
Residency year, *n* (%)			0.935
PGY-2	31 (62.0)	30 (60.0)	
PGY-3	19 (38.0)	20 (40.0)	
USMLE Step 2 CK score (mean ± SD)	248.3 ± 12.4	246.7 ± 13.8	0.542
In-training exam percentile (mean ± SD)	68.4 ± 18.2	66.9 ± 19.5	0.689
Prior respiratory elective, *n* (%)	6 (12.0)	7 (14.0)	0.766

### COPD grading accuracy outcomes

The primary outcome analysis revealed substantial differences in COPD grading accuracy between study groups at both assessment time points (Table 3). Immediately following the educational intervention, the intervention group achieved a mean accuracy rate of 86.4 ± 8.2% compared to 68.2 ± 12.3% in the control group, representing an 18.2 percentage point difference (95% CI, 14.1–22.3, *p* < 0.001). This large effect size (Cohen’s *d* = 1.74) persisted at the one-month follow-up assessment, with intervention group residents maintaining 82.6 ± 9.5% accuracy versus 64.8 ± 13.7% in controls (difference: 17.8, 95% CI: 13.2–22.4, *p* < 0.001).

**Table 3:** COPD grading accuracy rates at immediate and one-month assessments.

Assessment time point	Intervention group (*n* = 50)	Control group (*n* = 50)	Mean difference (95% CI)	*p*-value
Immediate post-intervention				
Overall accuracy, %	86.4 ± 8.2	68.2 ± 12.3	18.2 (14.1–22.3)	<0.001
Correct airflow limitation identification, %	94.8 ± 5.1	82.4 ± 9.8	12.4 (9.3–15.5)	<0.001
Correct GOLD grade assignment, %	84.2 ± 9.6	62.8 ± 14.2	21.4 (16.6–26.2)	<0.001
One-month follow-up				
Overall accuracy, %	82.6 ± 9.5	64.8 ± 13.7	17.8 (13.2–22.4)	<0.001
Correct airflow limitation identification, %	92.2 ± 6.3	78.6 ± 11.2	13.6 (10.1–17.1)	<0.001
Correct GOLD grade assignment, %	80.4 ± 10.8	58.2 ± 15.9	22.2 (17.0–27.4)	<0.001

**Figure 2:**
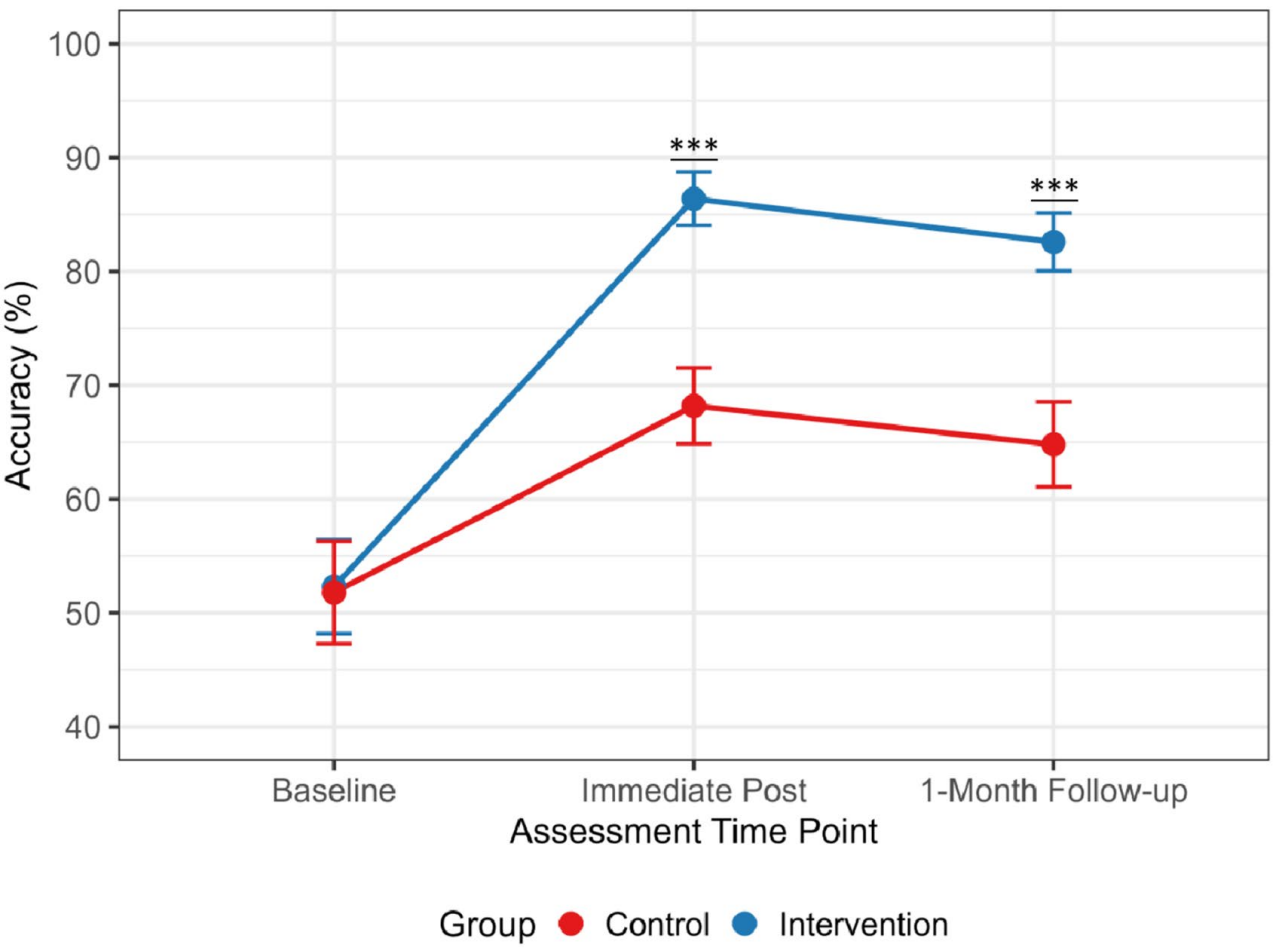
Depicting COPD grading accuracy rates over time for intervention and control groups, with error bars representing 95% confidence intervals. The line graph depicts the changes in overall COPD grading accuracy (%) for the intervention group (*n* = 50) and the control group (*n* = 50) at the immediate post-intervention and one-month follow-up assessments. The data were derived from residents’ performance on 10 standardized pulmonary function test cases. The intervention group demonstrated significantly higher accuracy at both time points (immediate: 86.4% vs. 68.2%; one-month: 82.6% vs. 64.8%; *p* < 0.001 for both between-group comparisons). ^***^p < 0.001.

**Figure 3:**
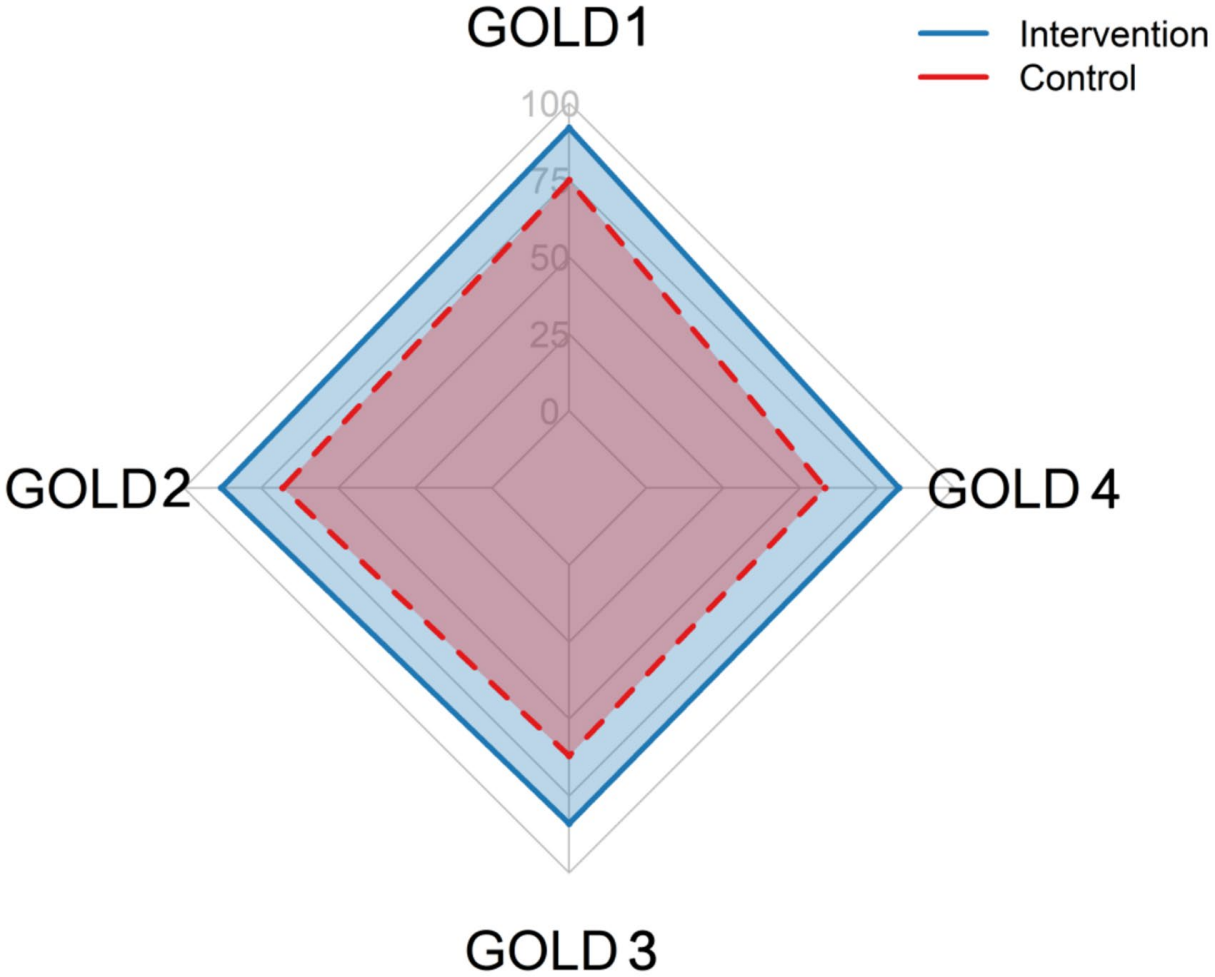
Comparing performance across different GOLD grade categories (A–D) between intervention and control groups at immediate assessment. This figure illustrates the accuracy of GOLD grade assignment by the intervention and control groups, broken down by the true severity (GOLD grades 1 through 4) of the 10 case-based evaluations administered immediately post-intervention. The data are derived from a sub-analysis of the primary outcome measure (COPD grading accuracy). Each bar represents the percentage of residents in each group who correctly identified the GOLD grade for cases of the specified true severity. The intervention group demonstrated superior performance across all severity levels, with the most substantial improvements observed for moderate and severe cases (GOLD 2 & 3).

Analysis of specific competency components revealed that the intervention group excelled particularly in identifying airflow limitation (FEV1/FVC < 0.70) and correctly assigning GOLD grades based on FEV1 percentage predicted values. The temporal pattern of accuracy changes differed between groups (Figure 2), with the intervention group showing only modest decline over the one-month period (3.8 percentage points) compared to a more substantial decrease in the control group (3.4 percentage points), though this group-by-time interaction did not reach statistical significance (*p* = 0.72). A detailed breakdown of grading accuracy by the underlying severity of the cases is presented in Figure 3, demonstrating the intervention group’s superior performance across all GOLD stages (1–4), with the most pronounced improvements observed for moderate and severe cases (GOLD 2 & 3).

### Comprehensive examination and self-efficacy outcomes

Secondary outcome analyses demonstrated consistent advantages for the intervention group across multiple domains (Table 4). Comprehensive examination scores were significantly higher in the intervention group (88.2 ± 6.4) compared to controls (78.6 ± 8.2), with a mean difference of 9.6 points (95% CI: 6.7–12.5, *p* < 0.001). Notably, the subset of examination items specifically addressing COPD-related content showed even larger between-group differences, with intervention group residents scoring 91.3 ± 5.8% versus 76.4 ± 10.2% for controls (*p* < 0.001).

**Table 4:** Secondary outcomes: examination scores, self-efficacy, and teaching satisfaction.

Outcome measure	Intervention group (*n* = 50)	Control group (*n* = 50)	Mean difference (95% CI)	*p*-value
Comprehensive examination				
Total score (0–100)	88.2 ± 6.4	78.6 ± 8.2	9.6 (6.7–12.5)	<0.001
COPD-specific items (%)	91.3 ± 5.8	76.4 ± 10.2	14.9 (11.6–18.2)	<0.001
Clinical reasoning items (%)	86.8 ± 7.2	75.2 ± 9.8	11.6 (8.2–15.0)	<0.001
Self-efficacy (SEPT scale)				
Overall score (1–5)	4.2 ± 0.5	3.4 ± 0.6	0.8 (0.6–1.0)	<0.001
PFT interpretation	4.4 ± 0.4	3.1 ± 0.7	1.3 (1.1–1.5)	<0.001
Clinical decision-making	4.1 ± 0.5	3.5 ± 0.6	0.6 (0.4–0.8)	<0.001
Patient communication	4.0 ± 0.6	3.6 ± 0.7	0.4 (0.1–0.7)	0.008
Teaching satisfaction				
Overall satisfaction (1–5)	4.6 ± 0.4	3.8 ± 0.7	0.8 (0.6–1.0)	<0.001
Content relevance	4.7 ± 0.4	4.0 ± 0.6	0.7 (0.5–0.9)	<0.001
Teaching method effectiveness	4.5 ± 0.5	3.5 ± 0.8	1.0 (0.7–1.3)	<0.001
Preparation for practice	4.4 ± 0.5	3.6 ± 0.7	0.8 (0.6–1.0)	<0.001

Self-efficacy assessments revealed particularly striking differences in residents’ confidence regarding pulmonary function test interpretation, with intervention group participants reporting mean scores of 4.4 ± 0.4 on the 5-point scale compared to 3.1 ± 0.7 for controls (*p* < 0.001). This enhanced confidence extended to broader clinical decision-making and patient communication domains, though effect sizes were somewhat smaller for these more general competencies.

### Teaching satisfaction and subgroup analyses

Teaching satisfaction ratings strongly favored the intervention approach across all evaluated dimensions (Table 5). Overall satisfaction scores averaged 4.6 ± 0.4 in the intervention group versus 3.8 ± 0.7 in controls (*p* < 0.001). Qualitative feedback highlighted the value of hands-on practice with authentic clinical materials, peer collaboration, and immediate expert feedback as key strengths of the flipped classroom approach.

**Table 5:** Distribution of teaching satisfaction ratings by study group.

Satisfaction level	Intervention group *n* (%)	Control group *n* (%)	*p*-value
Very satisfied (5)	32 (64.0)	12 (24.0)	<0.001
Satisfied (4)	16 (32.0)	18 (36.0)	
Neutral (3)	2 (4.0)	14 (28.0)	
Dissatisfied (2)	0 (0.0)	5 (10.0)	
Very dissatisfied (1)	0 (0.0)	1 (2.0)	

**Figure 4:**
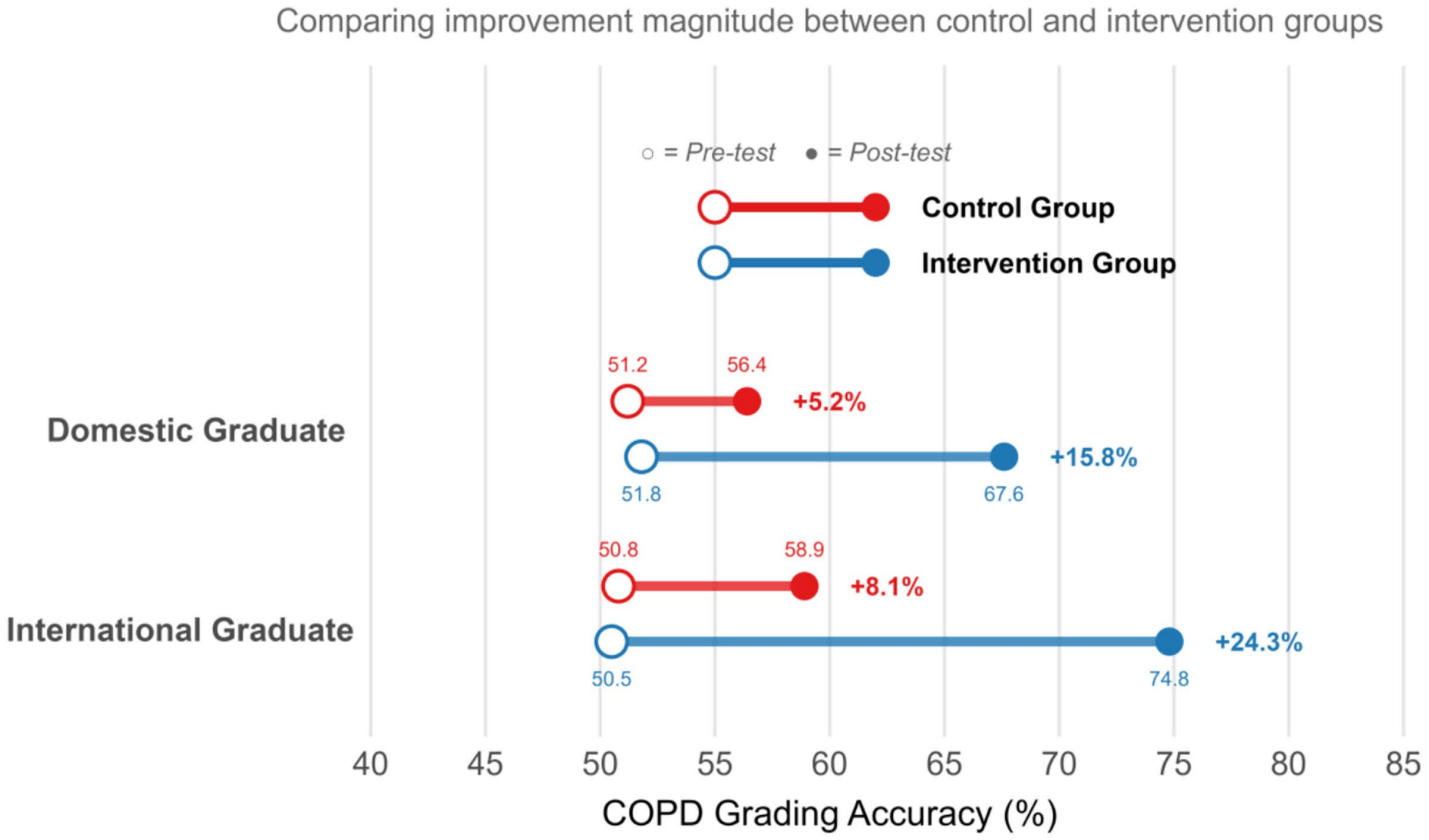
COPD grading accuracy improvements stratified by educational background (domestic vs. international medical graduates). The bar chart illustrates the mean improvement in COPD grading accuracy (percentage points) from baseline to the immediate post-intervention assessment, stratified by medical school background (Domestic vs. International graduates) for the intervention group (*n* = 50) and control group (*n* = 50). International medical graduates in the intervention group showed a particularly large benefit, with a mean improvement of 24.3 percentage points compared to 15.8 points for domestic graduates in the intervention group (interaction *p* = 0.042).

Subgroup analyses examining differential intervention effects based on learner characteristics revealed interesting patterns (Figure 4). International medical graduates showed particularly large benefits from the intervention approach, with accuracy improvements of 24.3 percentage points compared to 15.8 percentage points for domestic graduates (interaction *p* = 0.042). Similarly, residents with lower baseline academic performance (below median in-training examination scores) demonstrated greater relative improvements with the intervention, suggesting the approach may help level disparities in clinical competency development.

### Multivariable analysis results

Multivariable regression analysis confirmed the robust independent association between the educational intervention and improved COPD grading accuracy (Table 6). After adjusting for baseline characteristics such as age, gender, medical school background, baseline academic performance, and prior respiratory medicine exposure, participation in the flipped classroom intervention remained strongly associated with accuracy scores (*β* = 17.2, 95% CI: 13.8–20.6, *p* < 0.001). The model explained 58% of variance in grading accuracy (adjusted *R*^2^ = 0.58).

**Table 6:** Multivariable linear regression analysis of factors associated with COPD grading accuracy.

Variable	*β* coefficient	95% confidence interval	*p*-value
Educational intervention (intervention vs. control)	17.2	13.8–20.6	<0.001
Age	0.3	−0.5–1.1	0.452
Gender (female vs. male)	1.0	−2.3–4.3	0.558
Medical school background (international vs. domestic)	2.5	−1.0–6.0	0.164
Education level (MD/PhD or MD/MPH vs. MD only)	0.7	−2.6–4.0	0.679
Residency year (PGY-3 vs. PGY-2)	1.2	−2.1–4.5	0.475
USMLE Step 2 CK score	0.5	−0.2–1.2	0.156
In-training exam percentile	3.1	0.8–5.4	0.188
Prior respiratory elective (yes vs. no)	1.3	−2.5–5.1	0.503
Model statistics: adjusted *R*^2^ = 0.58; *p* < 0.001			

Propensity score matched analysis including 42 well-balanced pairs yielded consistent results, with the intervention group maintaining a 16.8 percentage point advantage in accuracy (95% CI: 12.4–21.2, *p* < 0.001). These findings demonstrate the robustness of the intervention effect across different analytical approaches and suggest that observed benefits are unlikely to be attributable to selection bias or unmeasured confounding.

## Discussion

This study provides compelling evidence that integrating paper-based pulmonary function test case discussions within a flipped classroom framework substantially enhances residents’ competency in COPD grading assessment. The observed 18-percentage point improvement in diagnostic accuracy represents a clinically meaningful advancement in resident education, with implications for patient care quality and safety. These findings contribute to the growing body of literature supporting innovative pedagogical approaches in medical education while addressing the specific challenge of developing complex clinical reasoning skills in respiratory medicine.

The magnitude of improvement observed in our intervention group aligns with and extends findings from recent systematic reviews of flipped classroom effectiveness in health professions education. Meta-analyses have consistently demonstrated superior learning outcomes with flipped classroom approaches, though effect sizes have varied considerably across studies and clinical domains (15, 16). Our results suggest that the combination of flipped classroom methodology with carefully structured case-based discussions using authentic clinical materials may amplify the benefits of either approach alone. The use of paper-based pulmonary function test reports, rather than digital simulations, appears to have facilitated deeper engagement with the material, as evidenced by sustained accuracy at one-month follow-up and enhanced self-efficacy scores.

Several mechanisms likely contribute to the effectiveness of our educational intervention. First, the pre-class preparation phase ensures that residents arrive with foundational knowledge of COPD pathophysiology and GOLD criteria, allowing face-to-face time to focus on higher-order application and analysis skills (17). This aligns with cognitive load theory, which suggests that managing intrinsic cognitive load through prior knowledge activation enables learners to devote greater resources to germane load associated with schema construction and automation (18). Second, the small group format with paper-based materials promotes active manipulation of data, collaborative problem-solving, and peer teaching—all elements associated with deeper learning and retention (19). The tactile nature of paper-based resources may also facilitate spatial reasoning and pattern recognition crucial for interpreting spirometry data and flow-volume loops.

The persistent benefits observed at one-month follow-up suggest that the intervention promotes durable learning rather than mere short-term performance gains. This finding is particularly noteworthy given the well-documented challenges of knowledge retention in medical education (20). The minimal decline in accuracy scores among intervention group participants (3.8 percentage points) compared to the steeper decline in controls indicates that the active learning strategies employed may facilitate better encoding and retrieval of clinical reasoning schemas. Furthermore, the enhanced self-efficacy scores in the intervention group likely contribute to continued engagement with pulmonary function test interpretation in clinical practice, creating a positive feedback loop that reinforces learning (21).

Our subgroup analyses revealing differential benefits for international medical graduates and lower-performing residents have important implications for educational equity. International medical graduates often face additional challenges in residency training, including differences in prior educational experiences and assessment methods (22). The structured, collaborative nature of our intervention appears to provide scaffolding that particularly benefits these learners, potentially by making implicit clinical reasoning processes more explicit and providing multiple opportunities for practice with feedback. Similarly, residents with lower baseline academic performance showed greater relative improvements, suggesting that the intervention may help address achievement gaps rather than simply benefiting already high-performing learners.

The superiority of the flipped classroom approach compared to traditional didactic teaching reflects fundamental differences in how clinical reasoning skills are developed. Traditional lectures, while efficient for content delivery, provide limited opportunity for active knowledge construction and application (23). In contrast, our intervention operationalizes principles of deliberate practice, providing repeated opportunities to engage with authentic clinical problems, receive immediate feedback, and refine diagnostic approaches (24). The collaborative aspect further enriches learning by exposing residents to diverse reasoning strategies and promoting metacognitive awareness through peer discussion.

A pertinent consideration is the potential confounding effect of differential instructional time and modality between the groups. The total structured learning time differed between the groups: the intervention group engaged in 3 h of structured pre-class study followed by a 90-min interactive in-class session, totaling 4.5 h; whereas the control group received 6 h of in-class lecture time with limited interactive elements and no structured pre-class preparation. It is recognized that didactic lectures are highly efficient for content delivery per unit time, potentially covering more breadth (25). In contrast, the flipped classroom model reallocates time to prioritize active, self-directed preparation and application-focused sessions. Evidence from cognitive load theory and health professions education indicates that while this approach might sacrifice some breadth, it leads to superior knowledge retention, deeper conceptual understanding, and enhanced skill transfer for complex clinical reasoning tasks (4, 26). Therefore, the observed outcomes likely result from this synergistic combination of modality and the quality of engaged time on task, underscoring that the primary goal of competency-based medical education is the achievement of durable learning outcomes.

Several limitations of this study warrant consideration. The retrospective design, while allowing analysis of a substantial cohort, introduces potential for selection bias and limits causal inference. Although propensity score matching and multivariable adjustment suggest robust findings, unmeasured confounders related to learner motivation or prior experiences may influence results. Furthermore, while the case-based assessments were standardized, the potential for evaluator bias cannot be entirely ruled out. Additionally, the total structured learning time differed between the intervention (4.5 h) and control (6 h) groups. This difference reflects the inherent nature of the flipped classroom model, which reallocates a portion of time for content delivery to self-directed pre-class study, thereby freeing up in-class time for active application. However, as detailed in Supplementary Table 1, the core learning objectives and content coverage regarding COPD pathophysiology, spirometry principles, and GOLD classification criteria were equivalent between groups. The observed outcomes are therefore more likely attributable to the differences in instructional methodology and the quality of engaged time rather than to a disparity in core content coverage. The single-center setting may limit generalizability, particularly to programs with different resident populations or resource constraints. Additionally, our follow-up period of 1 month, while demonstrating sustained benefits, does not address longer-term retention or transfer to clinical practice. Finally, the outcome measures were focused on assessment competency, knowledge, and self-efficacy; they do not establish whether the observed improvements translate into enhanced clinical behaviors or patient outcomes. Future prospective studies with extended follow-up and clinical outcome measures would strengthen the evidence base.

The resource implications of implementing our intervention deserve careful consideration. While the flipped classroom model requires substantial upfront investment in developing quality preparatory materials, these resources can be reused across multiple cohorts, improving cost-effectiveness over time (27). The use of paper-based materials, rather than expensive simulation technology, makes the approach accessible to programs with limited budgets. However, successful implementation requires faculty development to facilitate effective small group discussions and provide targeted feedback, representing an ongoing investment in educational quality.

Future research should explore several promising directions. First, investigating the optimal balance between digital and paper-based resources could refine the intervention while maintaining its effectiveness. Second, extending the approach to other complex diagnostic tasks in respiratory medicine, such as interpreting chest imaging or arterial blood gases, could broaden its impact. Third, examining the intervention’s effect on actual clinical outcomes, such as appropriate COPD management decisions or reduced diagnostic errors, would provide crucial validation of its real-world relevance. Finally, exploring how artificial intelligence tools might complement human instruction in providing personalized feedback on pulmonary function test interpretation represents an exciting frontier.

The implications of our findings extend beyond respiratory medicine education. As medical knowledge continues to expand exponentially, developing efficient methods for building complex clinical reasoning skills becomes increasingly critical (28). Our study demonstrates that thoughtfully designed educational interventions combining established pedagogical principles with authentic clinical materials can achieve substantial improvements in resident competency. The success of paper-based case discussions in an increasingly digital age also reminds us that the medium of instruction should be matched to learning objectives and cognitive demands rather than following technology trends uncritically.

## Conclusion

Integrating paper-based pulmonary function test case discussions into flipped classroom teaching significantly enhances residents’ COPD grading assessment competency, examination performance, and professional self-efficacy. This innovative pedagogical approach provides evidence-based guidance for respiratory medicine residency training programs. However, it should be noted that the present study is a single-center retrospective cohort design, and the generalizability of its findings may be limited by the specific characteristics of the participating institution’s residency program (e.g., resident background composition, training resource allocation). Therefore, it is recommended that future studies verify the intervention’s effectiveness in multi-center settings with diverse resident populations before broader implementation. When adopted in practice, programs should also tailor the intervention to their local educational contexts (e.g., adjusting pre-class material difficulty based on residents’ baseline knowledge) to maximize its educational impact. Overall, this teaching model holds promise for improving clinical reasoning skills related to COPD assessment and merits further exploration to confirm its long-term effects on resident clinical practice and patient outcomes.

## Data Availability

The original contributions presented in the study are included in the article/Supplementary material, further inquiries can be directed to the corresponding author.
