# Supplementary material for: Impact of integrating paper-based pulmonary function test case group discussions into flipped classroom on residents’ COPD grading assessment competency
Source: Front Med (Lausanne). 2026 Jan 7;12:1671616. doi: 10.3389/fmed.2025.1671616 (PMC12819828; doi:10.3389/fmed.2025.1671616)
Supplement: Supplementary file 1 [file Supplementary_file_1.docx]

# Supplementary Appendix: Pre-Class Video Lecture Self-Assessment Questions

*Corresponding to the 180-minute pre-class video lectures (COPD pathophysiology, spirometry principles, GOLD classification criteria) in the manuscript "Impact of Integrating Paper-Based Pulmonary Function Test Case Group Discussions into Flipped Classroom on Residents' COPD Grading Assessment Competency"*

## Overview

These self-assessment questions are embedded in the 180-minute pre-class video lectures (3 modules × 60 minutes each) to reinforce key knowledge and enable residents to self-monitor learning progress. Questions appear at the end of each video module, with immediate feedback provided after submission.

## Module 1: COPD Pathophysiology (60 minutes)

*Self-Assessment Questions (End of Module)*

### Instructions

Answer the following 5 questions to test your understanding of COPD pathophysiological mechanisms, phenotypes, and key pathological changes.

1. Which of the following best describes the **primary pathological feature** that distinguishes emphysema (a COPD phenotype) from chronic bronchitis?

A. Mucus gland hyperplasia in large airways

B. Alveolar wall destruction and loss of elastic recoil

C. Neutrophilic inflammation in small airways

D. Bronchial smooth muscle hypertrophy

1. In COPD, "air trapping" (increased residual volume) primarily results from which pathophysiological process?

A. Reduced lung compliance due to interstitial fibrosis

B. Increased airway resistance leading to incomplete exhalation

C. Pulmonary vasoconstriction causing right heart strain

D. Decreased tidal volume from respiratory muscle weakness

1. Which inflammatory cell is **most prominent** in the airways of patients with stable COPD and contributes to long-term disease progression?

A. Eosinophils

B. Neutrophils

C. Lymphocytes

D. Mast cells

1. What is the key consequence of "small airway remodeling" in COPD?

A. Increased airway diameter (reduced resistance)

B. Fixed narrowing of small airways (irreversible airflow limitation)

C. Enhanced bronchodilator responsiveness

D. Reduced mucus production

1. Why do patients with advanced COPD often develop "chronic respiratory acidosis" (pH < 7.35, PaCO₂ > 45 mmHg)?

A. Impaired gas exchange due to alveolar destruction

B. Reduced respiratory drive from chronic hypercapnia

C. Increased dead space ventilation (ventilation-perfusion mismatch)

D. All of the above

### Feedback for Module 1 Questions

1. **Correct Answer: B**

Rationale: Emphysema is defined by alveolar wall destruction and loss of elastic recoil, while chronic bronchitis is characterized by mucus gland hyperplasia (Option A) and small airway inflammation. Neutrophilic inflammation (Option C) is present in both phenotypes, and smooth muscle hypertrophy (Option D) is more typical of asthma.

1. **Correct Answer: B**

Rationale: Air trapping occurs when increased airway resistance (from narrowed small airways) slows exhalation, leaving air trapped in the lungs before the next inspiration. Interstitial fibrosis (Option A) is a restrictive lung disease feature; pulmonary vasoconstriction (Option C) causes cor pulmonale, not air trapping; respiratory muscle weakness (Option D) affects tidal volume but not air trapping directly.

1. **Correct Answer: B**

Rationale: Neutrophils are the dominant inflammatory cells in stable COPD, releasing proteases (e.g., elastase) that damage airways and alveoli. Eosinophils (Option A) are more common in asthma or COPD exacerbations; lymphocytes (Option C) contribute to chronic inflammation but are less prominent; mast cells (Option D) mediate acute bronchospasm (not long-term progression).

1. **Correct Answer: B**

Rationale: Small airway remodeling (thickening of airway walls, collagen deposition) causes fixed narrowing, leading to irreversible airflow limitation—the hallmark of COPD. It does not increase airway diameter (Option A), reduce bronchodilator responsiveness (Option C), or decrease mucus production (Option D).

1. **Correct Answer: D**

Rationale: Advanced COPD causes chronic respiratory acidosis via three mechanisms: (1) alveolar destruction impairs gas exchange (Option A); (2) chronic hypercapnia blunts respiratory drive (Option B); (3) increased dead space (ventilation to unperfused alveoli) worsens ventilation-perfusion mismatch (Option C).

## Module 2: Spirometry Principles (60 minutes)

*Self-Assessment Questions (End of Module)*

### Instructions

Answer the following 5 questions to test your understanding of spirometry equipment, key parameters, quality control, and normal/abnormal patterns.

1. Which spirometric parameter is **most critical** for diagnosing "obstructive airflow limitation" (the defining feature of COPD)?

A. Forced vital capacity (FVC)

B. Forced expiratory volume in 1 second (FEV₁)

C. FEV₁/FVC ratio

D. Peak expiratory flow (PEF)

1. To ensure valid spirometry results, what is the **minimum requirement** for acceptable test trials?

A. 1 trial with FEV₁ and FVC within 200 mL of each other

B. 2 acceptable trials with FEV₁ and FVC within 150 mL of each other

C. 3 acceptable trials with FEV₁ and FVC within 100 mL of each other

D. 4 trials (regardless of consistency)

1. A "flow-volume loop" with a **scalloped expiratory phase and prolonged exhalation** is characteristic of which type of lung disease?

A. Obstructive (e.g., COPD)

B. Restrictive (e.g., idiopathic pulmonary fibrosis)

C. Mixed (obstructive + restrictive)

D. Normal lung function

1. Why is **post-bronchodilator spirometry** mandatory for COPD diagnosis (per GOLD guidelines)?

A. To maximize FEV₁ values for severity grading

B. To distinguish COPD (minimal reversibility) from asthma (significant reversibility)

C. To reduce patient discomfort during forced exhalation

D. To confirm the presence of bronchospasm

1. Which factor **does not affect** the "predicted values" of FEV₁ and FVC (used to calculate FEV₁% predicted)?

A. Patient age

B. Patient height

C. Patient smoking history

D. Patient gender

### Feedback for Module 2 Questions

1. **Correct Answer: C**

Rationale: The FEV₁/FVC ratio is the gold standard for diagnosing obstructive airflow limitation—GOLD defines COPD as post-bronchodilator FEV₁/FVC < 0.70. FEV₁ (Option B) and FVC (Option A) alone are affected by age/height; PEF (Option D) is less reliable for COPD diagnosis.

1. **Correct Answer: B**

Rationale: Spirometry quality control requires at least 2 acceptable trials (with no technical errors) where FEV₁ and FVC values are within 150 mL of each other. Fewer trials (Option A) or larger variability (Options C/D) reduce result validity.

1. **Correct Answer: A**

Rationale: Obstructive lung diseases (e.g., COPD, asthma) cause a scalloped expiratory phase (due to airway narrowing) and prolonged exhalation (due to increased resistance). Restrictive diseases (Option B) show reduced FVC and normal FEV₁/FVC ratio; normal loops (Option D) have a smooth expiratory phase.

1. **Correct Answer: B**

Rationale: Post-bronchodilator spirometry assesses airflow reversibility—COPD shows minimal reversibility (FEV₁ improvement < 12% and < 200 mL), while asthma shows significant reversibility. It does not maximize FEV₁ for grading (Option A), reduce discomfort (Option C), or confirm bronchospasm (Option D).

1. **Correct Answer: C**

Rationale: Predicted FEV₁/FVC values are based on population norms adjusted for age (Option A), height (Option B), and gender (Option D). Smoking history (Option C) affects actual spirometric values (e.g., reduces FEV₁) but not the predicted reference values.

## Module 3: GOLD Classification Criteria (60 minutes)

*Self-Assessment Questions (End of Module)*

### Instructions

Answer the following 5 questions to test your understanding of GOLD spirometric grading, clinical grouping, symptom assessment, and exacerbation risk.

1. According to GOLD 2024, a patient with post-bronchodilator FEV₁% predicted = 42% falls into which **spirometric grade**?

A. GOLD 1 (Mild): FEV₁% predicted ≥ 80%

B. GOLD 2 (Moderate): 50% ≤ FEV₁% predicted < 80%

C. GOLD 3 (Severe): 30% ≤ FEV₁% predicted < 50%

D. GOLD 4 (Very Severe): FEV₁% predicted < 30%

1. The Modified Medical Research Council (mMRC) scale is used to assess which aspect of COPD in GOLD clinical grouping?

A. Spirometric severity

B. Dyspnea (shortness of breath) severity

C. Exacerbation risk

D. Comorbidity burden

1. A patient with GOLD 2 spirometry (FEV₁% predicted = 65%), mMRC score = 2 (dyspnea when climbing stairs), and 1 exacerbation in the past year falls into which **GOLD clinical group**?

A. Group A (Low risk, fewer symptoms)

B. Group B (Low risk, more symptoms)

C. Group C (High risk, fewer symptoms)

D. Group D (High risk, more symptoms)

1. Which of the following defines a "COPD exacerbation" (per GOLD)?

A. A gradual increase in cough and sputum over 2 weeks

B. An acute worsening of respiratory symptoms requiring additional treatment

C. A decline in FEV₁% predicted by ≥10% from baseline

D. Any episode of dyspnea requiring emergency department visit

1. For GOLD clinical grouping, "high risk" of exacerbations is defined by which criterion?

A. GOLD 3-4 spirometry OR ≥2 exacerbations in the past year

B. GOLD 2-4 spirometry OR ≥1 exacerbation in the past year

C. GOLD 1-2 spirometry AND ≥3 exacerbations in the past year

D. Only GOLD 4 spirometry (regardless of exacerbation history)

### Feedback for Module 3 Questions

1. **Correct Answer: C**

Rationale: GOLD 3 (Severe) is defined as post-bronchodilator FEV₁% predicted ≥30% and <50%. The patient’s 42% falls within this range—GOLD 2 (Option B) is 50-79%, GOLD 4 (Option D) is <30%.

1. **Correct Answer: B**

Rationale: The mMRC scale specifically grades dyspnea severity (e.g., mMRC 0 = no dyspnea; mMRC 4 = dyspnea at rest). Spirometric severity (Option A) uses FEV₁% predicted; exacerbation risk (Option C) uses spirometry and exacerbation history; comorbidities (Option D) are assessed separately.

1. **Correct Answer: B**

Rationale: GOLD Group B = Low risk (GOLD 1-2 spirometry + 0-1 exacerbations/year) + More symptoms (mMRC ≥2). The patient meets all criteria—Group A (Option A) requires mMRC 0-1; Groups C/D (Options C/D) require high risk (GOLD 3-4 or ≥2 exacerbations).

1. **Correct Answer: B**

Rationale: GOLD defines a COPD exacerbation as an acute worsening of respiratory symptoms (cough, sputum, dyspnea) that requires additional treatment (e.g., oral steroids, antibiotics). Gradual symptom increase (Option A) is stable disease; FEV₁ decline (Option C) and ED visits (Option D) are not required for diagnosis.

1. **Correct Answer: A**

Rationale: GOLD defines "high risk" as either (1) GOLD 3-4 spirometry (severe/very severe airflow limitation) OR (2) ≥2 exacerbations in the past year (regardless of spirometric grade). Options B/C/D incorrectly combine or restrict these criteria.
