# Supplementary material for: Impact of integrating paper-based pulmonary function test case group discussions into flipped classroom on residents’ COPD grading assessment competency
Source: Front Med (Lausanne). 2026 Jan 7;12:1671616. doi: 10.3389/fmed.2025.1671616 (PMC12819828; doi:10.3389/fmed.2025.1671616)
Supplement: Supplementary file 2 [file Supplementary_file_2.docx]

# Supplementary Appendix: Pre-Class Formative Assessment Questions (Flipped Classroom Intervention Group)

*Corresponding to the pre-class formative assessment (20 multiple-choice questions) described in the manuscript "Impact of Integrating Paper-Based Pulmonary Function Test Case Group Discussions into Flipped Classroom on Residents' COPD Grading Assessment Competency"*

## Instructions

- This assessment is designed to verify foundational knowledge of COPD pathophysiology, spirometry principles, and GOLD classification criteria before in-class sessions.
- Each question has one best answer.
- Score interpretation: ≥16/20 (80% correct) indicates mastery of prerequisite knowledge for face-to-face case discussions.

### 1. COPD Pathophysiology

Which of the following is the primary pathological change contributing to persistent airflow limitation in COPD?

A. Bronchial smooth muscle hypertrophy (predominant in asthma)

B. Alveolar wall destruction and small airway remodeling

C. Interstitial fibrosis of the lung parenchyma

D. Mucus gland hyperplasia in large central airways only

### 2. Spirometry Principles

What is the definition of "forced vital capacity (FVC)" in spirometry?

A. The maximum volume of air exhaled forcefully in the first second after a full inspiration

B. The total volume of air that can be exhaled forcefully from a full inspiration to complete expiration

C. The volume of air remaining in the lungs after a complete forced expiration

D. The ratio of FEV₁ to FVC, used to diagnose airflow limitation

### 3. GOLD Classification: Airflow Limitation

According to the 2024 GOLD guidelines, which parameter confirms "persistent airflow limitation" for COPD diagnosis?

A. Pre-bronchodilator FEV₁/FVC < 0.70

B. Post-bronchodilator FEV₁/FVC < 0.70

C. FEV₁% predicted < 80% (regardless of FEV₁/FVC ratio)

D. Presence of chronic cough and dyspnea (without spirometric confirmation)

### 4. COPD Pathophysiology

Which mechanism best explains the "dynamic hyperinflation" observed in patients with moderate-to-severe COPD?

A. Reduced lung compliance due to alveolar fibrosis

B. Increased airway resistance leading to incomplete exhalation before the next inspiration

C. Pulmonary vasoconstriction causing increased right ventricular afterload

D. Mucus plugging of large airways, reducing tidal volume

### 5. Spirometry Quality Control

To ensure valid spirometry results, which of the following is a critical technical requirement?

A. The patient should exhale slowly (over 10 seconds) to avoid airway collapse

B. The test must include at least 3 acceptable trials with FVC values within 150 mL of each other

C. The patient should inhale only to functional residual capacity (not total lung capacity)

D. Bronchodilator administration is optional for all patients

### 6. GOLD Spirometric Grading

A patient has post-bronchodilator FEV₁ = 1.8 L, FVC = 3.0 L, and FEV₁% predicted = 55%. What is their GOLD spirometric grade?

A. GOLD 1 (Mild): FEV₁% predicted ≥ 80%

B. GOLD 2 (Moderate): 50% ≤ FEV₁% predicted < 80%

C. GOLD 3 (Severe): 30% ≤ FEV₁% predicted < 50%

D. GOLD 4 (Very Severe): FEV₁% predicted < 30%

### 7. COPD Risk Factors

Which risk factor is the most modifiable and strongly associated with COPD development worldwide?

A. Occupational exposure to dust and chemicals

B. Cigarette smoking (including secondhand smoke)

C. Genetic factors (e.g., alpha-1 antitrypsin deficiency)

D. Indoor air pollution from biomass fuel use

### 8. Spirometry Interpretation

A 65-year-old male with a 40-pack-year smoking history has post-bronchodilator FEV₁/FVC = 0.68 and FEV₁% predicted = 72%. Which conclusion is correct?

A. No airflow limitation (FEV₁/FVC ≥ 0.70 is normal)

B. Confirmed airflow limitation, consistent with COPD

C. Borderline airflow limitation (needs repeat spirometry in 3 months)

D. Asthma (reversible airflow limitation, not COPD)

### 9. GOLD Symptom Assessment

The Modified Medical Research Council (mMRC) scale is used to evaluate which symptom in COPD patients?

A. Frequency of chronic cough

B. Severity of dyspnea (shortness of breath)

C. Volume of sputum production

D. Impact of COPD on quality of life

### 10. COPD Pathophysiology

Why do patients with advanced COPD often develop "cor pulmonale" (right heart failure)?

A. Hypoxic pulmonary vasoconstriction leading to increased pulmonary arterial pressure

B. Left ventricular dysfunction due to myocardial hypoxia

C. Fluid overload from reduced renal perfusion

D. Anemia-induced decreased oxygen-carrying capacity

### 11. Spirometry: Flow-Volume Loops

Which feature of a flow-volume loop is characteristic of obstructive lung disease (e.g., COPD)?

A. "Scalloped" expiratory phase with reduced peak flow and prolonged exhalation

B. "Flat" inspiratory phase due to upper airway obstruction

C. Reduced total lung capacity and vital capacity (restrictive pattern)

D. Normal peak flow but reduced FEV₁/FVC ratio

### 12. GOLD Comprehensive Assessment

A patient with GOLD 2 (Moderate) spirometric grade, mMRC score = 1 (mild dyspnea), and 0 exacerbations in the past year falls into which GOLD clinical group?

A. Group A (Low risk, fewer symptoms)

B. Group B (Low risk, more symptoms)

C. Group C (High risk, fewer symptoms)

D. Group D (High risk, more symptoms)

### 13. COPD Pathophysiology

What is the role of "neutrophilic inflammation" in COPD progression?

A. It reduces airway mucus production (protective effect)

B. It contributes to airway wall damage and alveolar destruction via proteases (e.g., elastase)

C. It increases bronchodilator responsiveness (beneficial for treatment)

D. It is absent in stable COPD (only present during exacerbations)

### 14. Spirometry: Bronchodilator Response

Why is a bronchodilator administered before spirometry for COPD diagnosis?

A. To assess reversibility of airflow limitation (distinguish COPD from asthma)

B. To maximize FVC and FEV₁ values for accurate grading

C. To reduce patient discomfort during forced exhalation

D. To confirm the presence of bronchospasm (required for COPD diagnosis)

### 15. GOLD Exacerbation Risk

Which patient has the highest risk of COPD exacerbations (per GOLD 2024)?

A. GOLD 1 spirometry, mMRC = 0, 1 exacerbation in 2 years

B. GOLD 2 spirometry, mMRC = 2, 0 exacerbations in 1 year

C. GOLD 3 spirometry, mMRC = 1, 2 exacerbations (1 hospitalized) in 1 year

D. GOLD 2 spirometry, mMRC = 3, 1 exacerbation in 1 year

### 16. COPD Differential Diagnosis

Which condition is most likely to be misdiagnosed as COPD (due to overlapping symptoms of dyspnea and cough)?

A. Idiopathic pulmonary fibrosis (restrictive lung disease)

B. Asthma (reversible obstructive lung disease)

C. Community-acquired pneumonia (acute infectious lung disease)

D. Pulmonary embolism (acute vascular disease)

### 17. Spirometry: Predicted Values

FEV₁% predicted is calculated by comparing a patient’s FEV₁ to:

A. The patient’s own baseline FEV₁ (from previous spirometry)

B. Age-matched, height-matched, and gender-matched healthy reference values

C. The patient’s FVC (to derive FEV₁/FVC ratio)

D. The maximum FEV₁ achievable with bronchodilator therapy

### 18. GOLD Treatment Principles

For a patient in GOLD Group B (Low risk, more symptoms), what is the first-line pharmacologic treatment per GOLD 2024?

A. Short-acting beta-agonist (SABA) monotherapy (as needed)

B. Long-acting muscarinic antagonist (LAMA) or long-acting beta-agonist (LABA) monotherapy

C. Inhaled corticosteroid (ICS) + LABA combination therapy

D. Triple therapy (ICS + LABA + LAMA)

### 19. COPD Pathophysiology

Which of the following is a key consequence of "alveolar destruction" in emphysema (a phenotype of COPD)?

A. Increased lung compliance (loss of elastic recoil)

B. Decreased residual volume (air trapping is reduced)

C. Increased diffusion capacity of the lung for carbon monoxide (DLCO)

D. Reduced airway resistance (airflow is improved)

### 20. Spirometry: Clinical Utility

Beyond diagnosing COPD, spirometry is also used to:

A. Predict life expectancy in all respiratory diseases

B. Monitor disease progression and response to treatment in COPD

C. Confirm the presence of pulmonary hypertension

D. Distinguish viral from bacterial COPD exacerbations

## Answer Key & Rationale

| Question Number | Correct Answer | Rationale |
| --- | --- | --- |
| 1 | B | Alveolar wall destruction (emphysema) and small airway remodeling (chronic bronchitis) are the core pathological changes causing irreversible airflow limitation in COPD. A is typical of asthma; C is restrictive lung disease; D is incorrect (mucus hyperplasia affects small airways too). |
| 2 | B | FVC is the total volume of air exhaled forcefully from full inspiration to complete expiration. A = FEV₁; C = residual volume; D = FEV₁/FVC ratio. |
| 3 | B | GOLD 2024 requires post-bronchodilator FEV₁/FVC < 0.70 to confirm persistent airflow limitation (pre-bronchodilator values may be affected by transient bronchospasm). |
| 4 | B | Dynamic hyperinflation occurs when increased airway resistance slows exhalation, leaving air trapped in the lungs before the next inspiration. A = restrictive disease; C = cor pulmonale; D = acute mucus plugging (not chronic hyperinflation). |
| 5 | B | Valid spirometry requires ≥3 acceptable trials with FVC/FEV₁ within 150 mL (to ensure consistency). A = incorrect (exhalation should be forceful); C = incorrect (inhale to total lung capacity); D = incorrect (bronchodilators are required for diagnosis). |
| 6 | B | GOLD 2 is defined as 50% ≤ FEV₁% predicted < 80%. The patient’s FEV₁% predicted = 55%, which falls into this range. |
| 7 | B | Cigarette smoking is the most modifiable and dominant risk factor for COPD globally (accounting for ~80-90% of cases). Other factors are less common or less modifiable. |
| 8 | B | Post-bronchodilator FEV₁/FVC < 0.70 confirms airflow limitation, consistent with COPD (even with mild FEV₁% predicted). C is incorrect (GOLD does not require repeat testing for ratios < 0.70). |
| 9 | B | The mMRC scale specifically grades dyspnea severity (e.g., mMRC 1 = dyspnea when walking faster than peers). Quality of life is assessed by the CAT scale. |
| 10 | A | Chronic hypoxia triggers pulmonary vasoconstriction, leading to increased pulmonary arterial pressure and right ventricular hypertrophy (cor pulmonale). B = left heart failure (unrelated to COPD); C = fluid overload (consequence, not cause); D = anemia (not linked to cor pulmonale). |
| 11 | A | Obstructive flow-volume loops show reduced peak flow, a "scalloped" expiratory phase, and prolonged exhalation. B = upper airway obstruction; C = restrictive pattern; D = incorrect (peak flow is reduced in COPD). |
| 12 | A | GOLD Group A = low risk (GOLD 1-2, 0-1 exacerbations/year) + fewer symptoms (mMRC 0-1). The patient meets all criteria. |
| 13 | B | Neutrophils release proteases (e.g., neutrophil elastase) that damage airway walls and alveoli, driving COPD progression. A = incorrect (neutrophils increase mucus); C = incorrect (reduces bronchodilator response); D = incorrect (present in stable COPD). |
| 14 | A | Bronchodilators help distinguish COPD (minimal reversibility) from asthma (significant reversibility). B = incorrect (FEV₁/FVC ratio, not absolute values, is key for diagnosis); C = incorrect (purpose is not comfort); D = incorrect (bronchospasm is not required for COPD diagnosis). |
| 15 | C | High risk = GOLD 3-4 spirometry or ≥2 exacerbations/year (1 hospitalized). The patient has GOLD 3 spirometry and 2 exacerbations (1 hospitalized), placing them at highest risk. |
| 16 | B | Asthma and COPD share symptoms (dyspnea, cough) and obstructive spirometric patterns; the key distinction is reversibility of airflow limitation. A = restrictive; C/D = acute (not chronic). |
| 17 | B | FEV₁% predicted compares a patient’s FEV₁ to population-based reference values (adjusted for age, height, gender, and race) to account for physiological differences. |
| 18 | B | GOLD 2024 recommends LAMA or LABA monotherapy as first-line for Group B (targets persistent symptoms). A = insufficient for persistent symptoms; C/D = reserved for higher-risk groups. |
| 19 | A | Alveolar destruction reduces elastic recoil, increasing lung compliance (lungs become "floppy"). B = incorrect (residual volume increases); C = incorrect (DLCO decreases); D = incorrect (airway resistance increases). |
| 20 | B | Spirometry monitors COPD progression (e.g., declining FEV₁) and treatment response (e.g., stable FEV₁ with bronchodilators). A = incorrect (cannot predict life expectancy); C = incorrect (echocardiogram diagnoses pulmonary hypertension); D = incorrect (sputum culture distinguishes infection type). |
