# Supplementary material for: Impact of integrating paper-based pulmonary function test case group discussions into flipped classroom on residents’ COPD grading assessment competency
Source: Front Med (Lausanne). 2026 Jan 7;12:1671616. doi: 10.3389/fmed.2025.1671616 (PMC12819828; doi:10.3389/fmed.2025.1671616)
Supplement: Supplementary file 3 [file Supplementary_file_3.docx]

# 10 Real Paper-Based Pulmonary Function Test Reports (English Version)

All reports adhere to the **ATS/ERS Technical Standards (2022)** and **Chinese Pulmonary Function Report Guidelines** (2021), including complete patient information, standardized spirometric data, flow-volume loops (annotated), and physician conclusions. Key parameters (FEV₁, FVC, FEV₁/FVC, RV/TLC, DLCO) align with GOLD criteria and clinical diagnostic standards for obstructive/restrictive lung diseases.

## Report 1: Chronic Obstructive Pulmonary Disease (GOLD 2)

### Basic Information

- **Pulmonary Function Test Request Form**

Institution: XX Hospital Respiratory Medicine Department

Medical Record No.: 20230401-056

Name: Zhang Jianguo

Gender: Male

Age: 65 Years

Height: 172.0 cm

Weight: 68.0 kg

Smoking History: 40 Pack-Years (20 cigarettes/day × 40 years, current smoker)

Clinical Diagnosis: COPD (Post-acute exacerbation)

Requested Tests: Pulmonary Ventilation + Lung Volumes + Diffusing Capacity

Ordering Physician: Li Na

Request Date: April 1, 2023

### Test Data (Table Format on Paper Report)

| Parameter | Measured Value | Predicted Value | % Predicted | Normal Range (% Predicted) |
| --- | --- | --- | --- | --- |
| Vital Capacity (VC) | 2.41 L | 3.62 L | 66.6% | 80–120% |
| Forced Vital Capacity (FVC) | 2.15 L | 3.45 L | 62.3% | 80–120% |
| Forced Expiratory Volume in 1s (FEV₁) | 1.32 L | 2.58 L | 51.2% | 80–120% |
| FEV₁/FVC Ratio | 61.4% | — | — | >70% |
| Maximum Voluntary Ventilation (MVV) | 38.5 L/min | 92.6 L/min | 41.6% | 80–120% |
| Residual Volume (RV) | 3.82 L | 2.85 L | 134.0% | 80–120% |
| Total Lung Capacity (TLC) | 6.23 L | 6.47 L | 96.3% | 80–120% |
| RV/TLC Ratio | 61.3% | — | — | <40% (Normal) |
| Diffusing Capacity for CO (DLCO) | 18.2 ml/(min·mmHg) | 27.5 ml/(min·mmHg) | 66.2% | 80–120% |

### Graph Annotations (Handwritten on Paper)

- **Flow-Volume Loop**: Expiratory phase shows a "scooped" depression (annotated: "Consistent with small airway obstruction"). Peak Expiratory Flow (PEF) = 4.2 L/s (below predicted 6.8 L/s).
- **Time-Volume Curve**: No plateau at end-expiration (annotated: "Incomplete exhalation, typical of obstructive defect").

### Test Conclusion (Physician’s Handwritten Signature)

1. Ventilatory function shows **moderate obstructive defect** (FEV₁ 51.2% predicted, FEV₁/FVC 61.4%), consistent with GOLD 2 COPD.
2. Elevated RV (134.0% predicted) and RV/TLC (61.3%) (annotated: "Air trapping due to emphysema").
3. Mild reduction in DLCO (66.2% predicted), indicating mild alveolar-capillary membrane impairment.
4. Recommendation: Bronchodilator reversibility test; long-term bronchodilator therapy.

- Technologist: Wang Hao
- Reviewing Physician: Liu Min
- Test Date: April 2, 2023

## Report 2: Bronchial Asthma (Positive Bronchodilator Test)

### Basic Information

- **Pulmonary Function Test Request Form**

Institution: XX Hospital Respiratory Medicine Department

Outpatient No.: 20230512-108

Name: Chen Yu

Gender: Female

Age: 32 Years

Height: 163.0 cm

Weight: 52.0 kg

Smoking History: None

Allergy History: Dust mite allergy

Clinical Diagnosis: Bronchial Asthma (Poorly controlled)

Requested Tests: Pulmonary Ventilation + Bronchodilator Test

Ordering Physician: Zhao Yang

Request Date: May 12, 2023

### Test Data (Pre- vs. Post-Bronchodilator)

| Parameter | Pre-Bronchodilator | Pre-% Predicted | Post-Bronchodilator | Post-% Predicted | Improvement Rate |
| --- | --- | --- | --- | --- | --- |
| FVC | 2.68 L | 89.3% | 2.85 L | 95.0% | 6.3% |
| FEV₁ | 1.82 L | 68.1% | 2.35 L | 87.7% | 29.1% |
| FEV₁/FVC | 67.9% | — | 82.5% | — | — |
| PEF | 5.1 L/s | 72.9% | 7.3 L/s | 104.3% | 43.1% |

### Graph Annotations (Handwritten Comparison)

- **Pre- vs. Post-Bronchodilator Flow-Volume Loops**: Pre-test expiratory depression resolved post-treatment (annotated: "Complete reversal of airway obstruction").
- **Time-Volume Curve**: Post-test FEV₁ plateau reached earlier (annotated: "Improved expiratory completeness").

### Test Conclusion

1. Pre-bronchodilator: Mild obstructive defect (FEV₁ 68.1% predicted, FEV₁/FVC 67.9%).
2. Post-bronchodilator: FEV₁ improvement rate = 29.1% (>12% + 200 mL), **positive bronchodilator test**—consistent with bronchial asthma.
3. Recommendation: Inhaled corticosteroid-LABA combination therapy; avoid allergen exposure.

- Technologist: Sun Jia
- Reviewing Physician: Zhao Yang
- Test Date: May 12, 2023

## Report 3: Idiopathic Pulmonary Fibrosis (Restrictive Defect)

### Basic Information

- **Pulmonary Function Test Request Form**

Institution: XX Hospital Respiratory Medicine Department

Inpatient No.: 20230620-091

Name: Li Ming

Gender: Male

Age: 58 Years

Height: 175.0 cm

Weight: 65.0 kg

Smoking History: 10 Pack-Years (quit 15 years ago)

Clinical Diagnosis: Suspected Idiopathic Pulmonary Fibrosis (IPF)

Requested Tests: Pulmonary Ventilation + Lung Volumes + DLCO

Ordering Physician: Wang Wei

Request Date: June 20, 2023

### Test Data

| Parameter | Measured Value | Predicted Value | % Predicted | Normal Range |
| --- | --- | --- | --- | --- |
| VC | 1.85 L | 3.92 L | 47.2% | 80–120% |
| FVC | 1.78 L | 3.75 L | 47.5% | 80–120% |
| FEV₁ | 1.52 L | 3.02 L | 50.3% | 80–120% |
| FEV₁/FVC | 85.4% | — | — | >70% |
| TLC | 4.21 L | 7.15 L | 58.9% | 80–120% |
| RV | 1.95 L | 3.10 L | 62.9% | 80–120% |
| RV/TLC | 46.3% | — | — | <40% |
| DLCO | 9.8 ml/(min·mmHg) | 26.3 ml/(min·mmHg) | 37.3% | 80–120% |

### Graph Annotations

- **Flow-Volume Loop**: Narrow "stubby" shape (annotated: "Reduced vital capacity, typical of restrictive defect").
- **Chest CT Correlation**: Annotated: "Subpleural reticulation + honeycombing (from CT report 20230618), consistent with IPF."

### Test Conclusion

1. Ventilatory function shows **severe restrictive defect** (TLC 58.9% predicted, VC 47.2% predicted).
2. Severe reduction in DLCO (37.3% predicted), indicating diffuse alveolar-capillary damage.
3. Consistent with IPF-related interstitial lung disease.
4. Recommendation: Pulmonary rehabilitation; antifibrotic therapy evaluation.

- Technologist: Chen Xi
- Reviewing Physician: Wang Wei
- Test Date: June 21, 2023

## Report 4: Normal Pulmonary Function

### Basic Information

- **Pulmonary Function Test Request Form**

Institution: XX Hospital Health Check-Up Center

Health Record No.: 20230705-122

Name: Wang Ting

Gender: Female

Age: 28 Years

Height: 165.0 cm

Weight: 55.0 kg

Smoking History: None

Clinical Diagnosis: Routine health check-up (no respiratory symptoms)

Requested Tests: Basic Pulmonary Ventilation

Ordering Physician: Zhang Li

Request Date: July 5, 2023

### Test Data

| Parameter | Measured Value | Predicted Value | % Predicted | Normal Range |
| --- | --- | --- | --- | --- |
| FVC | 3.25 L | 3.01 L | 108.0% | 80–120% |
| FEV₁ | 2.78 L | 2.45 L | 113.5% | 80–120% |
| FEV₁/FVC | 85.5% | — | — | >70% |
| PEF | 8.2 L/s | 7.5 L/s | 109.3% | 80–120% |
| FEF₂₅₋₇₅ (Max Mid-Expiratory Flow) | 6.1 L/s | 5.8 L/s | 105.2% | 80–120% |

### Graph Annotations

- **Flow-Volume Loop**: Smooth expiratory phase, no depression; PEF peak sharp (annotated: "Normal effort, no obstruction").
- **Time-Volume Curve**: Plateau reached within 6 seconds (annotated: "Complete exhalation, normal ventilation").

### Test Conclusion

1. All ventilatory parameters are within normal range (FEV₁ 113.5% predicted, FEV₁/FVC 85.5%).
2. No evidence of obstructive or restrictive lung disease.
3. Recommendation: Annual routine follow-up.

- Technologist: Li Jia
- Reviewing Physician: Zhang Li
- Test Date: July 5, 2023

## Report 5: Severe COPD (GOLD 4) with Cor Pulmonale

### Basic Information

- **Pulmonary Function Test Request Form**

Institution: XX Hospital Respiratory Medicine Department

Medical Record No.: 20230810-077

Name: Zhao Daming

Gender: Male

Age: 72 Years

Height: 168.0 cm

Weight: 60.0 kg

Smoking History: 55 Pack-Years (quit 3 years ago)

Clinical Diagnosis: Severe COPD (GOLD 4) + Cor Pulmonale

Requested Tests: Ventilation + Lung Volumes + DLCO + SpO₂ Monitoring

Ordering Physician: He Lin

Request Date: August 10, 2023

### Test Data

| Parameter | Measured Value | Predicted Value | % Predicted | Normal Range |
| --- | --- | --- | --- | --- |
| FVC | 1.32 L | 3.25 L | 40.6% | 80–120% |
| FEV₁ | 0.68 L | 2.35 L | 28.9% | 80–120% |
| FEV₁/FVC | 51.5% | — | — | >70% |
| TLC | 7.85 L | 6.95 L | 112.9% | 80–120% |
| RV | 5.23 L | 2.95 L | 177.3% | 80–120% |
| RV/TLC | 66.6% | — | — | <40% |
| DLCO | 7.2 ml/(min·mmHg) | 24.8 ml/(min·mmHg) | 29.0% | 80–120% |
| Resting SpO₂ | 88% (room air) | — | — | >95% |

### Graph Annotations

- **Flow-Volume Loop**: Severe expiratory depression; PEF = 2.1 L/s (annotated: "Severe small airway obstruction").
- **Time-Volume Curve**: No plateau (annotated: "Severe air trapping; test terminated early due to dyspnea").

### Test Conclusion

1. **Very severe obstructive defect** (FEV₁ 28.9% predicted, FEV₁/FVC 51.5%)—consistent with GOLD 4 COPD.
2. Marked air trapping (RV 177.3% predicted, RV/TLC 66.6%) and severe DLCO reduction (29.0% predicted).
3. Resting hypoxemia (SpO₂ 88% on room air)—consistent with cor pulmonale.
4. Recommendation: Long-term oxygen therapy (LTOT); avoid respiratory infections.

- Technologist: Zhao Qian
- Reviewing Physician: He Lin
- Test Date: August 11, 2023

## Report 6: Bronchiectasis with Mild Obstruction

### Basic Information

- **Pulmonary Function Test Request Form**

Institution: XX Hospital Respiratory Medicine Department

Outpatient No.: 20230915-045

Name: Liu Fang

Gender: Female

Age: 45 Years

Height: 160.0 cm

Weight: 50.0 kg

Smoking History: None

Clinical Diagnosis: Bronchiectasis (right lower lobe)

Requested Tests: Pulmonary Ventilation + Bronchodilator Test

Ordering Physician: Sun Tao

Request Date: September 15, 2023

### Test Data

| Parameter | Pre-Bronchodilator | Pre-% Predicted | Post-Bronchodilator | Post-% Predicted | Improvement Rate |
| --- | --- | --- | --- | --- | --- |
| FVC | 2.52 L | 92.5% | 2.60 L | 95.6% | 3.2% |
| FEV₁ | 1.98 L | 78.4% | 2.05 L | 81.2% | 3.5% |
| FEV₁/FVC | 78.6% | — | 78.8% | — | — |
| FEF₂₅₋₇₅ | 4.2 L/s | 75.0% | 4.3 L/s | 76.8% | 2.4% |

### Graph Annotations

- **Flow-Volume Loop**: Mild expiratory depression at mid-phase (annotated: "Mild small airway obstruction, consistent with bronchiectasis").
- **CT Correlation**: Annotated: "Right lower lobe bronchiectasis (CT report 20230910) — localized airflow limitation."

### Test Conclusion

1. Pre-bronchodilator: **Mild obstructive defect** (FEV₁ 78.4% predicted, FEF₂₅₋₇₅ 75.0% predicted).
2. Negative bronchodilator test (FEV₁ improvement rate = 3.5% < 12%)—fixed airway obstruction from bronchiectasis.
3. Recommendation: Airway clearance therapy; antibiotic prophylaxis for exacerbations.

- Technologist: Wu Ying
- Reviewing Physician: Sun Tao
- Test Date: September 15, 2023

## Report 7: Obesity-Related Restrictive Defect

### Basic Information

- **Pulmonary Function Test Request Form**

Institution: XX Hospital Respiratory Medicine Department

Outpatient No.: 20231022-068

Name: Chen Wei

Gender: Male

Age: 40 Years

Height: 170.0 cm

Weight: 110.0 kg

BMI: 37.7 kg/m² (Class II Obesity)

Smoking History: None

Clinical Diagnosis: Dyspnea on exertion (obesity-related)

Requested Tests: Pulmonary Ventilation + Lung Volumes

Ordering Physician: Zhou Xin

Request Date: October 22, 2023

### Test Data

| Parameter | Measured Value | Predicted Value | % Predicted | Normal Range |
| --- | --- | --- | --- | --- |
| VC | 2.85 L | 4.02 L | 70.9% | 80–120% |
| FVC | 2.78 L | 3.85 L | 72.2% | 80–120% |
| FEV₁ | 2.42 L | 3.15 L | 76.8% | 80–120% |
| FEV₁/FVC | 87.1% | — | — | >70% |
| TLC | 5.95 L | 7.50 L | 79.3% | 80–120% |
| RV | 2.55 L | 3.20 L | 79.7% | 80–120% |
| RV/TLC | 42.9% | — | — | <40% |

### Graph Annotations

- **Flow-Volume Loop**: Narrow shape, normal expiratory phase (annotated: "Reduced volume due to chest wall restriction from obesity").
- **Clinical Note**: Annotated: "Dyspnea improves with weight loss (10 kg in 3 months) — no evidence of intrinsic lung disease."

### Test Conclusion

1. **Mild restrictive defect** (TLC 79.3% predicted, VC 70.9% predicted) — secondary to obesity-related chest wall restriction.
2. No obstructive defect (FEV₁/FVC 87.1%).
3. Recommendation: Weight management; aerobic exercise training.

- Technologist: Zheng Jie
- Reviewing Physician: Zhou Xin
- Test Date: October 23, 2023

## Report 8: Post-Lung Resection (Left Upper Lobe)

### Basic Information

- **Pulmonary Function Test Request Form**

Institution: XX Hospital Thoracic Surgery Department

Medical Record No.: 20231130-089

Name: Yang Guo

Gender: Male

Age: 55 Years

Height: 178.0 cm

Weight: 72.0 kg

Smoking History: 30 Pack-Years (quit after surgery)

Clinical Diagnosis: Post-left upper lobe resection (lung adenocarcinoma, pT1bN0M0)

Requested Tests: Pulmonary Ventilation + DLCO

Ordering Physician: Xu Feng

Request Date: November 30, 2023

### Test Data

| Parameter | Measured Value | Predicted Value (Post-Resection Adjusted) | % Predicted | Normal Range |
| --- | --- | --- | --- | --- |
| FVC | 3.12 L | 3.65 L | 85.5% | 80–120% |
| FEV₁ | 2.58 L | 2.95 L | 87.5% | 80–120% |
| FEV₁/FVC | 82.7% | — | — | >70% |
| DLCO | 21.5 ml/(min·mmHg) | 26.8 ml/(min·mmHg) | 80.2% | 80–120% |

### Graph Annotations

- **Flow-Volume Loop**: Symmetric shape, slightly reduced peak flow (annotated: "Compensatory function adequate post-resection").
- **Surgical Note**: Annotated: "Left upper lobe resection (June 2023) — no evidence of airflow limitation or diffusion impairment."

### Test Conclusion

1. Ventilatory function and DLCO are within normal range (adjusted for post-lung resection).
2. Adequate pulmonary compensation; no functional impairment.
3. Recommendation: Post-surgical pulmonary rehabilitation; annual oncology follow-up.

- Technologist: Han Lei
- Reviewing Physician: Xu Feng
- Test Date: December 1, 2023

## Report 9: Bronchiectasis (Bilateral Lower Lobes) with Moderate Obstruction

### Basic Information

- **Pulmonary Function Test Request Form**

Institution: XX Hospital Respiratory Medicine Department

Outpatient No.: 20231215-039

Name: Zhang Lina

Gender: Female

Age: 52 Years

Height: 158.0 cm

Weight: 48.0 kg

Smoking History: None

Clinical Diagnosis: Bronchiectasis (bilateral lower lobes) + Recurrent pneumonia

Requested Tests: Pulmonary Ventilation + Lung Volumes + Sputum Culture Correlation

Ordering Physician: Chen Min

Request Date: December 15, 2023

### Test Data

| Parameter | Measured Value | Predicted Value | % Predicted | Normal Range (% Predicted) |
| --- | --- | --- | --- | --- |
| Vital Capacity (VC) | 2.12 L | 3.25 L | 65.2% | 80–120% |
| Forced Vital Capacity (FVC) | 1.98 L | 3.08 L | 64.3% | 80–120% |
| Forced Expiratory Volume in 1s (FEV₁) | 1.25 L | 2.42 L | 51.7% | 80–120% |
| FEV₁/FVC Ratio | 63.1% | — | — | >70% |
| Max Mid-Expiratory Flow (FEF₂₅₋₇₅) | 3.0 L/s | 5.6 L/s | 53.6% | 80–120% |
| Total Lung Capacity (TLC) | 5.02 L | 5.85 L | 85.8% | 80–120% |
| Residual Volume (RV) | 2.90 L | 2.60 L | 111.5% | 80–120% |
| RV/TLC Ratio | 57.8% | — | — | <40% (Normal) |

### Graph Annotations (Handwritten on Paper)

- **Flow-Volume Loop**: Expiratory phase shows moderate "scooping" (annotated: "Consistent with small airway obstruction, typical of bronchiectasis"). FEF₂₅₋₇₅ reduced (3.0 L/s) — annotated: "Early indicator of small airway involvement."
- **Clinical Correlation**: Annotated: "Sputum culture (+) for *Haemophilus influenzae* (12/14/2023) — aligns with infectious exacerbation of bronchiectasis."

### Test Conclusion (Physician’s Handwritten Signature)

1. Ventilatory function shows **moderate obstructive defect** (FEV₁ 51.7% predicted, FEV₁/FVC 63.1%), consistent with bilateral lower lobe bronchiectasis.
2. Elevated RV (111.5% predicted) and RV/TLC (57.8%) — indicative of air trapping from chronic airway dilation.
3. FEF₂₅₋₇₅ reduction confirms small airway dysfunction, a key feature of bronchiectasis.
4. Recommendation: Antibiotic therapy for *H. influenzae* infection; airway clearance techniques (e.g., chest physiotherapy); long-term bronchodilator use.

- Technologist: Li Xiao
- Reviewing Physician: Chen Min
- Test Date: December 16, 2023

## Report 10: Post-Viral Pneumonia Obstructive Defect (Recovering Phase)

### Basic Information

- **Pulmonary Function Test Request Form**

Institution: XX Hospital Respiratory Medicine Department

Medical Record No.: 20240120-062

Name: Wang Hao

Gender: Male

Age: 38 Years

Height: 176.0 cm

Weight: 70.0 kg

Smoking History: 5 Pack-Years (quit 2 years ago)

Clinical Diagnosis: Post-viral pneumonia (COVID-19, 3 months prior) + Persistent dyspnea

Requested Tests: Pulmonary Ventilation + Bronchodilator Test

Ordering Physician: Zhao Yan

Request Date: January 20, 2024

### Test Data (Pre- vs. Post-Bronchodilator)

| Parameter | Pre-Bronchodilator | Pre-% Predicted | Post-Bronchodilator | Post-% Predicted | Improvement Rate |
| --- | --- | --- | --- | --- | --- |
| FVC | 3.52 L | 88.0% | 3.65 L | 91.3% | 3.7% |
| FEV₁ | 2.68 L | 76.6% | 2.92 L | 83.4% | 8.9% |
| FEV₁/FVC | 76.1% | — | 80.0% | — | — |
| PEF | 7.2 L/s | 82.8% | 7.9 L/s | 90.8% | 9.7% |
| FEF₂₅₋₇₅ | 4.8 L/s | 73.8% | 5.3 L/s | 81.5% | 10.4% |

### Graph Annotations (Handwritten Comparison)

- **Pre- vs. Post-Bronchodilator Flow-Volume Loops**: Pre-test mild expiratory depression partially resolved post-treatment (annotated: "Partial reversibility of airway obstruction — consistent with recovering post-viral inflammation").
- **Time-Volume Curve**: Post-test FEV₁ plateau reached faster (annotated: "Improved expiratory efficiency, suggesting resolution of residual bronchial inflammation").

### Test Conclusion

1. Pre-bronchodilator: **Mild obstructive defect** (FEV₁ 76.6% predicted, FEV₁/FVC 76.1%) — likely secondary to residual airway inflammation from post-COVID-19 pneumonia.
2. Post-bronchodilator: FEV₁ improvement rate = 8.9% (partial reversibility, <12% threshold) — indicates ongoing but resolving airway dysfunction.
3. Recommendation: Short-course bronchodilator therapy; pulmonary rehabilitation to restore full ventilatory function; follow-up PFT in 3 months.

- Technologist: Sun Wei
- Reviewing Physician: Zhao Yan
- Test Date: January 20, 2024
