# Supplementary material for: Impact of integrating paper-based pulmonary function test case group discussions into flipped classroom on residents’ COPD grading assessment competency
Source: Front Med (Lausanne). 2026 Jan 7;12:1671616. doi: 10.3389/fmed.2025.1671616 (PMC12819828; doi:10.3389/fmed.2025.1671616)
Supplement: Supplementary file 4 [file Table_1.docx]

**Supplementary Table 1. Comparison of Learning Objectives, Instructional Content, and Time Allocation Between Study Groups**

| **Component** | **Intervention Group**  **(Flipped Classroom with Case Discussion)** | **Control Group (Traditional Didactic Lecture)** | **Parity in Core Content** |
| --- | --- | --- | --- |
| **Learning Objectives** | 1. Explain COPD pathophysiology and GOLD criteria. 2. Accurately interpret PFTs and assign GOLD grades. 3. Develop clinical reasoning skills through case analysis. | 1. Explain COPD pathophysiology and GOLD criteria. 2. Accurately interpret PFTs and assign GOLD grades. | Identical for core knowledge & interpretation (Obj 1 & 2) |
| **Pre-class/Self-Study** | **Duration: 3 hours** Content: Video lectures , readings, interactive modules, formative assessments on COPD pathophysiology, spirometry, GOLD criteria. | **Duration: Not formally assigned** Content: General reading recommendations only (no structured materials). | Core knowledge delivered in intervention group's pre-class work was covered in control group's lectures. |
| **In-class/Formal Session** | **Duration: 1.5 hours** Content: Structured small-group discussion of 8-10 paper-based PFT cases. Focus on application, collaborative interpretation, and faculty-facilitated feedback. | **Duration: 6 hours** Content: Didactic lectures covering COPD epidemiology, pathophysiology, diagnosis, management, and PFT interpretation (via projected examples). | Core knowledge (Obj 1 & 2) delivered in control lectures was covered in intervention group's pre-class work. Application focus (Obj 3) was unique to intervention in-class. |
| **Total Structured Time** | **4.5 hours** | **6 hours** | Similar, with a difference of 1.5 hours |
| **Primary Pedagogical Focus** | Active learning, knowledge application, collaborative reasoning, feedback on performance. | Passive knowledge acquisition, content delivery. | Different |
